# Analysis of the Effect of *Plutella xylostella Polycalin* and *ABCC2* Transporter on Cry1Ac Susceptibility by CRISPR/Cas9-Mediated Knockout

**DOI:** 10.3390/toxins15040273

**Published:** 2023-04-06

**Authors:** Lei Xiong, Zhaoxia Liu, Jingge Li, Shuyuan Yao, Zeyun Li, Xuanhao Chen, Lingling Shen, Zhen Zhang, Yongbin Li, Qing Hou, Yuhang Zhang, Minsheng You, Zhiguang Yuchi, Shijun You

**Affiliations:** 1 State Key Laboratory of Ecological Pest Control for Fujian and Taiwan Crops, Institute of Applied Ecology, Fujian Agriculture and Forestry University, Fuzhou 350002, China; 2 Ministerial and Provincial Joint Innovation Centre for Safety Production of Cross-Strait Crops, Fujian Agriculture and Forestry University, Fuzhou 350002, China; 3 Joint International Research Laboratory of Ecological Pest Control, Ministry of Education, Fuzhou 350002, China; 4 College of Oceanology and Food Science, Quanzhou Normal University, Quanzhou 362000, China; 5 School of Pharmaceutical Science and Technology, Tianjin University, Tianjin 300072, China

**Keywords:** polycalin, ABCC2, *Bacillus thuringiensis*, CRISPR/Cas9

## Abstract

Many insects, including the *Plutella xylostella* (L.), have developed varying degrees of resistance to many insecticides, including *Bacillus thuringiensis* (*Bt*) toxins, the bioinsecticides derived from *Bt*. The polycalin protein is one of the potential receptors for *Bt* toxins, and previous studies have confirmed that the Cry1Ac toxin can bind to the polycalin protein of *P. xylostella*, but whether polycalin is associated with the resistance of *Bt* toxins remains controversial. In this study, we compared the midgut of larvae from Cry1Ac-susceptible and -resistant strains, and found that the expression of the *Pxpolycalin* gene was largely reduced in the midgut of the resistant strains. Moreover, the spatial and temporal expression patterns of *Pxpolycalin* showed that it was mainly expressed in the larval stage and midgut tissue. However, genetic linkage experiments showed that the *Pxpolycalin* gene and its transcript level were not linked to Cry1Ac resistance, whereas both the *PxABCC2* gene and its transcript levels were linked to Cry1Ac resistance. The larvae fed on a diet containing the Cry1Ac toxin showed no significant change in the expression of the *Pxpolycalin* gene in a short term. Furthermore, the knockout of *polycalin* and ATP-binding cassette transporter subfamily C2 *(ABCC2)* genes separately by CRISPR/Cas9 technology resulted in resistance to decreased susceptibility to Cry1Ac toxin. Our results provide new insights into the potential role of polycalin and ABCC2 proteins in Cry1Ac resistance and the mechanism underlying the resistance of insects to *Bt* toxins.

## 1. Introduction

*Bacillus thuringiensis* (*Bt*) is a kind of gram-positive bacterium which is friendly to people and the environment [1]. The active insecticidal ingredient produced by *Bt* can be used to control many kinds of insect pests, including lepidopteran, coleopteran, dipteran, etc [2]. However, with the long-term use of *Bt* toxins, a large number of insects have evolved resistance to *Bt* [3,4], including diamondback moth, *Plutella xylostella* (L.), the first reported case of *Bt* resistance in the field [5]. There is a pressing need to develop resistance management strategies accordingly.

Understanding the action mechanism of the *Bt* protein and insect resistance mechanism to *Bt* is of great significance for pest control and sustainable use of GM crops. Currently, the mechanism models of *Bt* mainly include the sequential binding model [6,7] and signal transduction pathway [7,8], among which the binding of toxin and receptor proteins is undoubtedly a key link for *Bt* toxins to play a role in. In addition, Guo et al. [9,10,11] found that the MAPK signaling pathway regulates the *Bt* resistance of *P. xylostella* by regulating the differential expression of ALP, APN, and ABCC genes, while MAPK [10,12] is regulated by the juvenile hormone (JH) [13] and 20-hydroxyecdysone (20E). At present, *Bt* toxin receptors have been reported mainly including aminopeptidase (APN) [14,15,16,17,18], cadherin/cadherin-like (CAD) [19,20,21,22,23,24,25], alkaline phosphatase (ALP) [26,27] and ATP binding cassette (ABC) transporter [28,29,30,31,32,33,34,35,36,37]. With the development of further research, an increasing number of receptor proteins, genes, and resistance pathways [38,39,40] have been reported to be involved in the toxicity of *Bt* to insects and the resistance of insects to *Bt* toxins, including polycalin in this study.

In 2016, Mauchamp et al. [41] identified a new lipid carrier protein in silkworm for the first time by two-dimensional electrophoresis and mass spectrometry, and named it polycalin (Pentadecacalin), where polycalin is a term coined [41] to describe proteins with multiple, lipocalin-like domains. Polycalin proteins of *Bombyx mori* [42], *Helicoverpa armigera* [43,44,45,46,47], *P. xylostella*, [48] and *Spodoptera exigua* [49] were demonstrated to bind to *Bt* Cry toxin by mass spectrometry or in vitro binding experiments. Similarly, heterologously expressed polycalin of *H. armigera* [44] showed a high affinity for the Cry2Aa toxin, and the mortality of neonate larvae decreased by 31.84% after ingestion of polycalin antisera and the Cry1Ac toxin. Using Western blot and Ligand blot experiments, Zhan et al. [50] first confirmed that the polycalin protein of *P. xylostella* has the property of binding to the Cry1Ac toxin, and the addition of Pxpolycalinf to *Sf9* cells expressing ATP-binding cassette transporter subfamily C2 (ABCC2) significantly increased the toxicity of Cry1Ac to cells [51]. As a potential receptor for the Cry1Ac toxin in *P. xylostella*, the role of *Pxpolycalin* in *Bt* resistance in vivo remains to be studied.

In this study, we found that the expression level of the *Pxpolycalin* gene in the Cry1Ac-resistant strain was significantly lower than that in the Cry1Ac-susceptible strain, and the transcription level of the *Pxpolycalin* gene did not change significantly in a short term after feeding on the *Bt* Cry1Ac toxin. However, genetic linkage experiments showed that the *Pxpolycalin* gene and its transcript level were not linked to Cry1Ac resistance, whereas both the *PxABCC2* gene and its transcript levels were linked to Cry1Ac resistance. The *Pxpolycalin* and *PxABCC2* homozygous mutant strains were obtained using the CRISPR/Cas9 system, respectively, and bioassay experiments showed that these two homozygous mutant strains resulted in increased resistance and decreased susceptibility to the Cry1Ac toxin, indicating the possibility that *Pxpolycalin* and *PxABCC2* genes are involved in Cry1Ac resistance. Further studies are needed to determine how they are involved in the resistance of *P. xylostella*. These findings will advance our understanding of *polycalin* function and contribute to the development of strategies for managing insect resistance.

## 2. Results

### 2.1. Pxpolycalin Sequence Comparison between P. xylostella Strains

**Figure 1:**
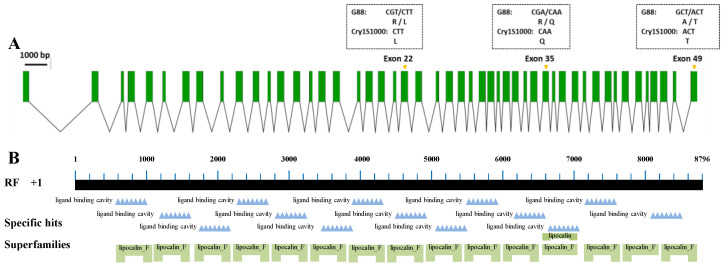
Structure of *Pxpolycalin* gene in *P. xylostella*. (**A**) Genomic structure of the *Pxpolycalin* gene in *P. xylostella*. Green boxes indicate the exons, and the spaces between the two boxes indicate the introns. The figure is drawn to scale, and the corresponding scale bar is shown. The dotted boxes above exons 22, 35, and 49 are fixed site mutations between G88 and Cry1S1000 strains. (**B**) NCBI conserved domain database (CDD)-based annotation of the *Pxpolycalin* gene sequence.

**Figure 2:**
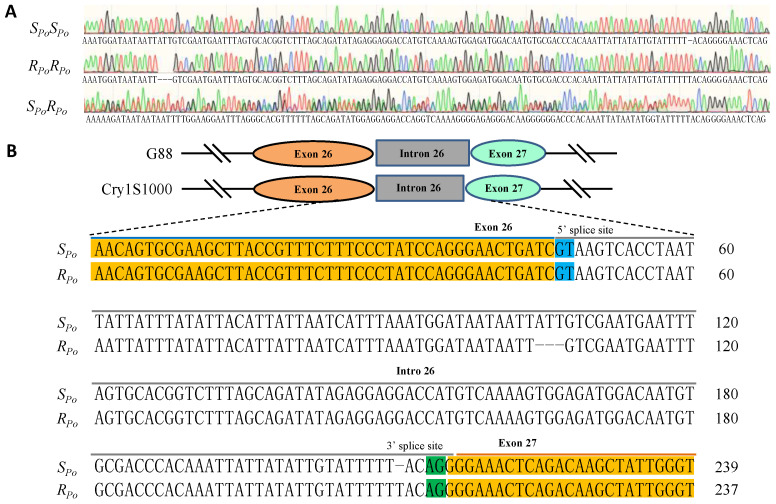
*R_Po_* mutation of *Pxpolycalin* from the Cry1S1000 strain. (**A**) The difference in the genotype of *Pxpolycalin* between the G88 and Cry1S1000 strains based on PCR and sequencing. *S_Po_S_Po_* is the sequencing result of the G88 strain, *R_Po_R_Po_* is the sequencing result of the Cry1S1000 strain, and *S_Po_R_Po_* is the sequencing result of hybrid F_1_ between G88 and Cry1S1000 strains. (**B**) Alignment of gDNA sequences of the *S_Po_* allele and the *R_Po_* allele. Sequences highlighted in orange are exons 26 and 27.

Full-length *Pxpolycalin* transcripts were amplified by PCR and sequenced with two pairs of specific primers using a midgut cDNA template generated from the Cry1Ac-susceptible strain (G88) and Cry1Ac-resistant strain (Cry1S1000). After sequencing, the results of gene cloning were spliced and an 8829 bp (OP381323) fragment was obtained and encoded a predicted 2942 aa protein. Between the susceptible and resistant strains, we found a total of three fixed amino acid variations, which were R/L1255L, R/Q2097Q, and A/T2942T (Figure 1A) between the two strains. Our study found that there were two types of amino acids at these three sites in eight randomly selected larvae of the G88 strain, but only one amino acid was selected and retained at the same site of the Cry1S1000 strain, and they were distributed in exons 22, 35 and 49 (Figure 1A). Domain analysis showed that the *Pxpolycalin* gene contained 14 ligand binding sites, 1 lipocalin site, and 15 lipocalin-like structure (Figure 1B), and the second point mutation (R/Q2097Q) was on the ligand binding site. In addition, using sequence amplification and sequencing techniques, we amplified gDNA fragments specific for *Pxpolycalin* and *PxABCC2* genes in G88 and Cry1S1000 strains (Figure 2 and Figure S1).

### 2.2. Phylogenetic Analysis of Pxpolycalin in P. xylostella

**Figure 3:**
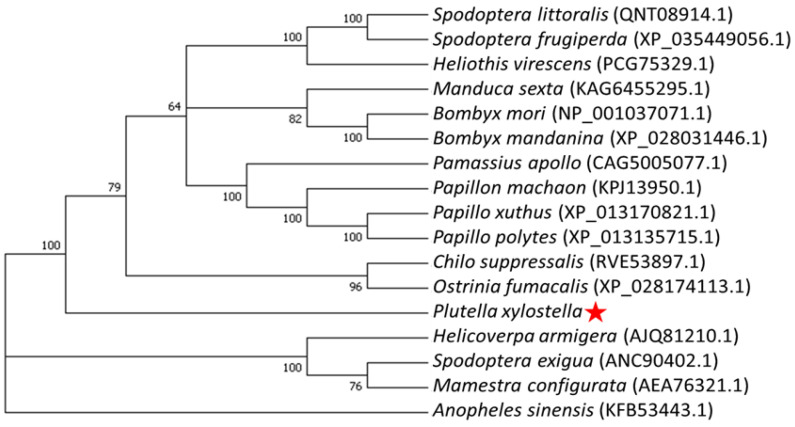
Neighbor-joining tree based on the amino acid sequences of Pxpolycalin in *P. xylostella* and other insects. The scale bar indicates the phylogenetic distance (bootstrap = 1000). The polycalin protein of *P. xylostella* is represented by a red asterisk.

The amino acid sequence encoded by Pxpolycalin was used to construct a phylogenetic tree with polycalin proteins of 16 other insect species. Referring to the bootstrap of 1000 replications, Pxpolycalin clustered with the existing polycalin orthologues in lepidoptera but was more distantly related to insects such as *H. armigera*, *S. exigua*, and *Mamestra configurata* (Figure 3). In addition, it can be found from Figure 3 that Pxpolycalin was the most distantly related to *Anopheles Sinensis* of Diptera and they belong to two distinct clades entirely. Based on this, we hypothesized that the polycalin of *P. xylostella*, similar to the polycalin of other Lepidopteran insects, might also be the receptor protein of the *Bt* Cry toxin.

### 2.3. Spatio-Temporal Expression Patterns of the Pxpolycalin Gene in G88 and Cry1S1000 Strains

**Figure 4:**
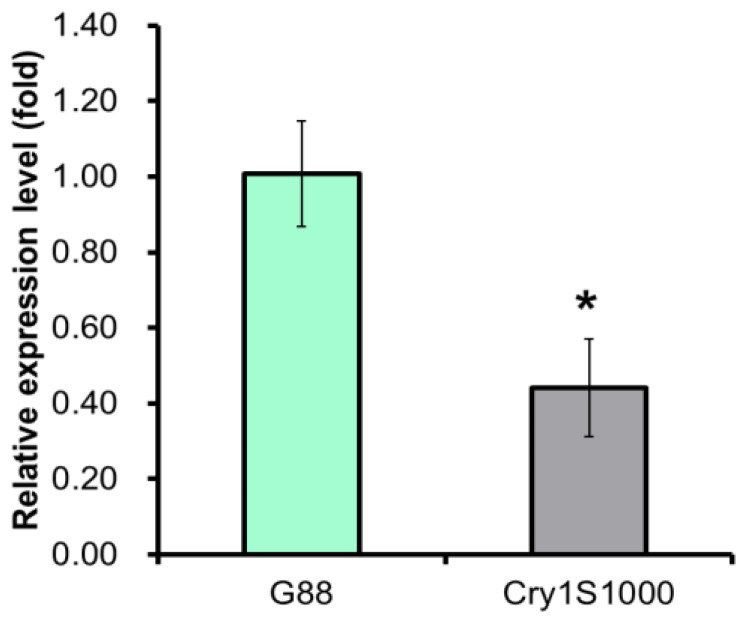
Relative *polycalin* transcription detected by qRT-PCR in G88 susceptible and Cry1S1000 resistant larvae of *P. xylostella*. Asterisks (*) indicate significant difference for *p* < 0.05. The data were evaluated by Student’s *t* test using SPSS v.26.0.

**Figure 5:**
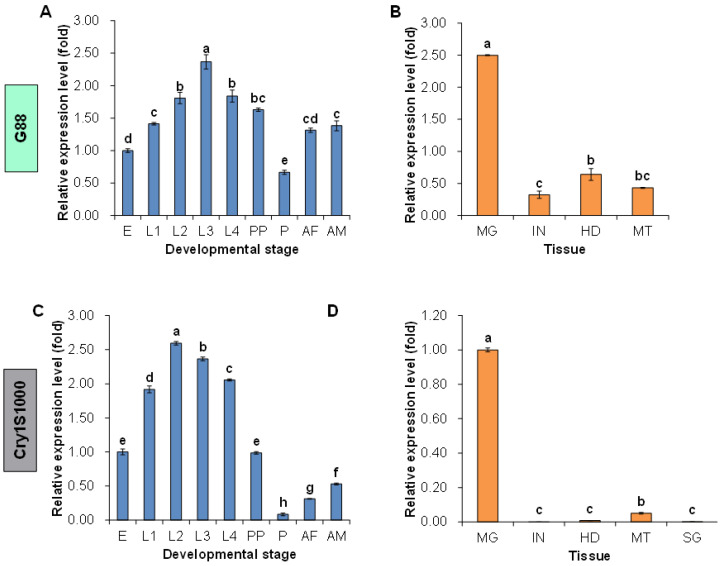
Relative expression level of *Pxpolycalin* in different developmental stages and different tissues of *P. xylostella*. (**A**,**B**) Relative expression level of *Pxpolycalin* in G88 strain; (**C**,**D**) Relative expression level of *Pxpolycalin* in Cry1S1000 strain. E: egg; L1: 1st instar larvae; L2: 2nd instar larvae; L3: 3rd instar larvae; 4th instar larvae; PP: prepupae; P: pupae; AF: female adults; AM: male adults. MG: midgut; IN: integument; HD: head; MT: malpighian tubules; SG: silk gland. Expression level was calculated according to the value of the egg (E) or midgut (MG), which was given an arbitrary value of 1. Data was represented with three biological replicates and each replication was repeated three times. The bars were shown as mean ± SD. Different letters above the bars indicate significant differences in different development stages or different tissues. Statistically significant differences were analyzed with one-way ANOVA (Tukey’s test for multiple comparisons, *p* < 0.05).

The qRT-PCR (qPCR) was conducted to explore the relative expression patterns of the *Pxpolycalin* gene in the midgut of the third instar larvae, different developmental stages, and different larva tissues with specific primers in the conserved region. The results showed a higher relative expression level in the larvae midgut of the G88-susceptible strain than in the Cry1S1000-resistant strain (Figure 4), implicating a potential connection between the *Pxpolycalin* gene and the resistance to *Bt* toxins. The developmental stages expression profile showed that the *Pxpolycalin* gene was expressed in almost all developmental stages collected, among which the susceptible strain had the highest expression at the third instar, while the resistant strain at the second instar and both strains had the lowest expression at the pupal stage, followed by the adults. In contrast, the expression level of this gene in the larval stage was about twice that in the adult stage, and the fold difference was higher in the resistant strain (Figure 5A,C). Similarly, analysis of expression patterns in different larval tissues showed that the *Pxpolycalin* gene was highly expressed mainly in the midgut, but almost unexpressed or expressed very little in other tissues, such as integument, head, malpighian tubules, and silk glands, especially in resistant strains (Figure 5B,D).

### 2.4. Induction of PxABCC2 and Pxpolycalin by Cry1Ac Toxin in G88 Strain

**Figure 6:**
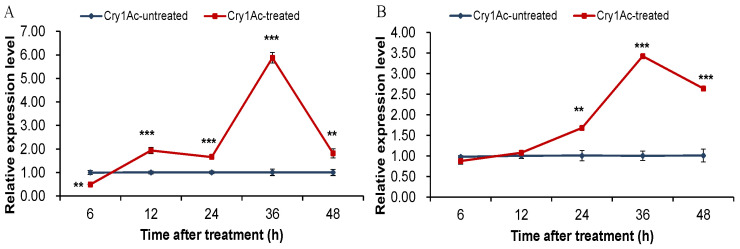
Effect of LC_10_ of Cry1Ac on the relative expression of *PxABCC2* (**A**) and *Pxpolycalin* (**B**) in the 3rd instar *P. xylostella* of the G88 strain. The results are shown as the mean ± SD. Asterisks above error bars represent significant differences (**, *p* < 0.01; ***, *p* < 0.001) using Student’s *t*-test.

Transcription levels of *PxABCC2* and *Pxpolycalin* genes in the G88 susceptible strain increased significantly from 6 to 36 h and reached a peak at 36 h after Cry1Ac toxin treatment. Among these, a transient reduction in transcript levels exists for both genes due to induction treatment of the Cry1Ac toxin. These results indicated that feeding on Cry1Ac toxin could inhibit the expression of *PxABCC2* and *Pxpolycalin* genes transitorily, which further supports that these two genes may be involved in resistance to the *Bt* Cry1Ac toxin in *P. xylostella* (Figure 6).

### 2.5. Genetic Linkage Analysis of the Pxpolycalin and PxABCC2 Genes

Since that we identified a 3 bp deletion and 1 bp insertion in the Cry1S1000 strain (Figure 2 and Figure S1), to test the linkage between the *Pxpolycalin* and Cry1Ac resistance, we designed specific primers to amplify the gDNA fragment containing the mutation to distinguish the susceptible-allele (*S_Po_S_Po_*) and resistant-allele (*R_Po_R_Po_*) genotypes. Using specific primers, we also amplified the gDNA of the *PxABCC2* gene by PCR in both strains. Their lengths (479 bp/552 bp) were significantly different, which allowed us to identify susceptible (*S_A2_S_A2_*) and resistant (*R_A2_R_A2_*) strains by genotypes. F_1_ progeny produced by crossing male-resistant with female-susceptible strains was then backcrossed with resistant strain to produce backcross families a and b. Although the genotypes of *R_Po_R_Po_*:*R_Po_S_Po_* were detected in a near 1:1 in the without-Cry1Ac-selected backcross families a and b, genotype *R_Po_S_Po_* was also detected in the Cry1Ac-selected group (Table 1), suggesting that the *Pxpolycalin* gene mutation was not linked to Cry1Ac resistance. Genotyping of 24 without-Cry1Ac-selected adults from the backcross family a and b revealed that the ratio of *R_A2_R_A2_*:*R_A2_S_A2_* among them was close to a 1:1 random separation ratio, including 14:10 (Fisher’s exact test, *χ^2^* = 11.46, *df* = 1, *p* < 0.001) for backcross family a and 15:9 (Fisher’s exact test, *χ^2^* = 12.86, *df* = 1, *p* < 0.000) for backcross family b (Table S1), indicating that there is co-segregation between the *PxABCC2* gene mutation and Cry1Ac resistance, that is, there is a genetic linkage relationship.

Similarly, we also investigated the co-segregation between *Pxpolycalin* and *PxABCC2* genes’ expression levels and Cry1Ac resistance by genetic linkage. Firstly, F_1_ progeny and backcross family a/b were obtained and selected on an artificial diet with or without a diagnostic dose of the Cry1Ac toxin. The expression level of the *Pxpolycalin* gene was not significantly co-segregated in both backcross families, with-Cry1Ac-selected and without-Cry1Ac-selected (Figure S2A), suggesting that the *Pxpolycalin* gene expression level was also not linked to Cry1Ac resistance. The qPCR results showed that the expression level of *PxABCC2* in F_1_ larvae was similar to that of the G88 susceptible strain, indicating that the resistance of Cry1S1000 was recessive. However, the expression levels of backcross families a and b with-Cry1Ac-selected or without-Cry1Ac-selected showed two distinct groups. Among the two backcross families, one group had a significantly reduced expression level similar to Cry1S1000, while the other group had an 8:12 (Fisher’s exact test, *χ^2^* = 10.00, *df* = 1, *p* < 0.05) separation ratio (Figure S2B), indicating a tight genetic linkage between *PxABCC2* and Cry1Ac resistance in *P. xylostella*.

### 2.6. Mutagenesis of Pxpolycalin and PxABCC2 Mediated by CRISPR/Cas9

**Figure 7:**
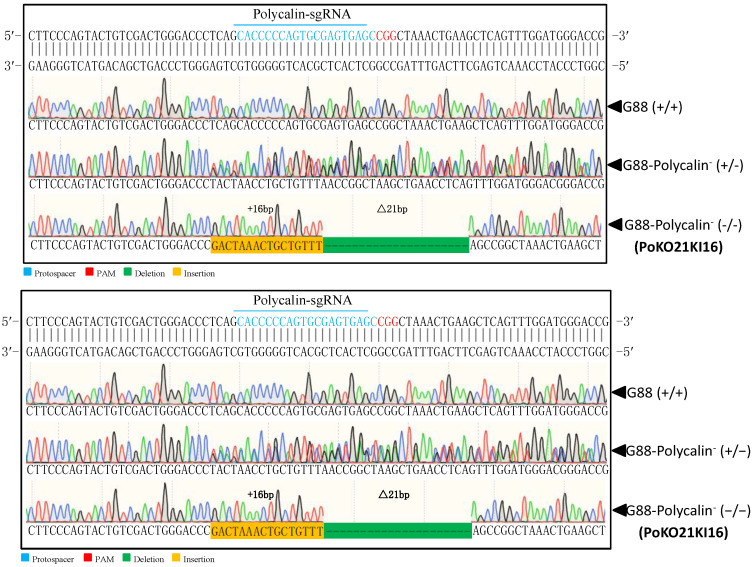
Mutagenesis of *Pxpolycalin* induced by CRISPR/Cas9. Partial sequences from the G88 and homozygous *Pxpolycalin* mutant showing the indels at the target sequence (polycalin-sgRNA) in exon 2 of *Pxpolycalin*.

To knock out *Pxpolycalin* and *PxABCC2*, a mixture of sgRNA and Cas9 protein was injected into fresh eggs laid in less than 15 min from the G88 strain, in which the homozygous mutant of *PxABCC2* (A3KO28) (Figure S3) was derived from the work of Liu et al., [31], and the *PxABCC2* mutant strain has been stored in the laboratory since 2020. For *Pxpolycalin*, only one homozygous mutant strain with a 16 bp insertion and 21 bp deletion in exon 2 was generated (PoKO21KI16) (Figure 7).

### 2.7. Effect of Pxpolycalin and PxABCC2 Mutations on the Susceptibility of Cry1Ac Toxin in P. xylostella

**Figure 8:**
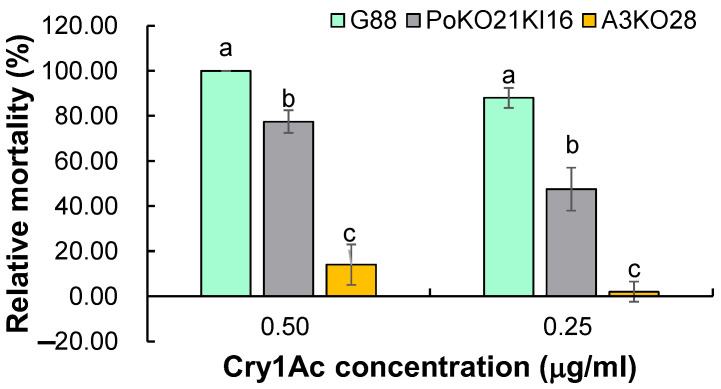
Relative mortality of different types of mutant homozygous strains after Cry1Ac toxin treatment. G88 indicates the Cry1Ac-susceptible strain. PoKO21KI16 indicates the homozygous strain for the *Pxpolycalin* mutation. A3KO28 indicates the homozygous strain for the *PxABCC2* mutation. The bars were shown as mean ± SD. Different letters above the bars indicate significant differences in different types of mutant homozygous strains. Statistically significant differences were analyzed with one-way ANOVA (Tukey’s test for multiple comparisons, *p* < 0.05).

To determine the susceptibility of *Pxpolycalin* and *PxABCC2* genes mutations to Cry1Ac toxin, we used two concentrations (0.50 and 0.25 μg/mL) to bioassay two single homozygous mutant strains (PoKO21KI16 and A3KO28). The results indicated that mutations in these two genes led to different degrees of resistance to Cry1Ac toxin in *P. xylostella* larvae. Specifically, the mortality rate of the A3KO28 strain (14.00 ± 2.00%) was significantly lower than that of the PoKO21KI16 strain (77.55 ± 5.00%), and the mortality of both strains was significantly lower than that of wild type (G88) (Figure 8). The bioassay results suggest that *Pxpolycalin* and *PxABCC2* genes may play a role in the resistance of *P. xylostella* to the Cry1Ac toxin.

## 3. Discussion

The mechanism of action of *Bt* Cry toxins and the resistance mechanism of insects to *Bt* are both very complex processes. At present, the two commonly accepted models are sequential binding model [7,52,53] and the signal transduction model [7,8,54], among which the binding of *Bt* toxins to the receptor in insect midgut is undoubtedly considered to be the key to its action. Up to now, several insect midgut proteins, such as cadherin [8,20,55], aminopeptidase N [18,56,57], alkaline phosphatase [27,58] and ABC transporter [29,32,59,60,61], have been reported to act as receptors for *Bt* toxins in the process of *Bt* action. The polycalin protein involved in this study has been shown to bind to Cry1Ac [43,51] and Cry2Aa [44] toxins in vitro in previous studies, which indicates that it may also act as a receptor protein similar to other known receptors. Since the binding of the protein to the toxin does not prove that the protein is the receptor protein of the toxin [51], the role of the polycalin protein in the resistance of *P. xylostella* to *Bt* remains to be further studied.

Studies have shown that structural mutations of receptor proteins or changes in gene expression levels can be the cause of insect resistance to *Bt*. For example, Xiao et al. [29] showed that the wrong cleavage of *HaABCC2* was related to the resistance of *H. armigera* to *Bt* toxins. The study of Liu et al. [31] also found that there were more ABCC2 transcripts in the Cry1S1000 resistance of *P. xylostella*, and the double mutation of *PxABCC2* and *PxABCC3* led to a high level of resistance to the Cry1Ac toxin in *P. xylostella* larvae. In addition, some studies have shown that changes in the expression of *ABCH1* and *ABCG* genes are related to *Bt* resistance [9,62]. In this study, we found a few amino acid variants in susceptible and resistant strains, and it is still unknown whether they play a role in *Bt* resistance. Subsequently, the relationship between this amino acid sites and *Bt* resistance can be verified by site-directed mutagensis. In addition, we also found fixed variants in the intron portion of the *Pxpolycalin* gene and the *PxABCC2* gene between G88-susceptible and Cry1S1000-resistant strains, providing us with a simple method to distinguish susceptible and resistant strains by PCR amplification. In previous studies [31], we have confirmed that the *PxABCC2* gene of the Cry1S1000 resistant strain has multiple alternative splicing patterns, among which different mutation types lead to the premature translation termination of the PxABCC2 protein. Therefore, we speculate that the transcription error may lead to the generation of resistance. Unfortunately, no significant changes in sequence length were found in the eight individuals of resistant strain amplified by us. Whether the differences in intron length of the *Pxpolycalin* gene contribute to the development of resistance or whether the *Pxpolycalin* gene is involved in *Bt* resistance in some other way needs to be further studied.

The expression level of the *Pxpolycalin* gene in the susceptible strain was significantly higher than that in the resistant strain. In addition, after treating *P. xylostella* larvae with the Cry1Ac toxin, it was found that the expression level of the *Pxpolycalin* gene was transiently inhibited by the Cry1Ac toxin in a short period, but the expression level of the *Pxpolycalin* gene increased with the increase of larval instar. In this regard, the *Pxpolycalin* gene has the characteristics of being a potential receptor for the *Bt* Cry1Ac toxin.

The overexpression and inhibition of receptor proteins are commonly used to study the role of *Bt* toxins binding proteins in the mode of action of *Bt* toxins. For example, overexpression of the *PxABCC2* gene in *Drosophila melanogaster* results in increased susceptibility to the *Bt* Cry1Ac toxin [63]. Overexpression of the *SlABCC2* gene in Sf9 cells also increased the toxicity of Cry1Ca toxin to cells [33]. The overexpression of *ABCG10*, *ABCH3*, and *ABCH4* in *Aphis craccivora* promoted the tolerance to imidacloprid [61]. In addition, downregulation of the *PxABCB1* gene expression significantly reduced the susceptibility of *P. xylostella* larvae to the Cry1Ac toxin [64]. The susceptibility of *Leptinotarsa decemlineata* larvae to the Cry3Aa toxin was reduced by silencing the expression of the ABC transporter using RNAi technology [65]. Silencing of *CsABCC2* also significantly reduced the susceptibility of the Cry1C toxin [66]. Furthermore, the knockdown of the *APN* gene also reduced the susceptibility to Cry1Ab, Cry1Ac, and Cry1Ca toxins [18]. Silencing the expression of the *HzALP2* gene in *Helicoverpa zea* larvae can improve the survival rate of larvae in a diet containing the Cry1Ac toxin [27]. CRISPR/Cas9-mediated mutation of ABC transporter *ABCA2* induces different degrees of resistance to the Cry2Ab toxin in *Trichoplusia ni* [67] and *Pectinophora gossypiella* [68]. A homozygous mutation of the *OfCad* gene generated by CRISPR/Cas9 caused the *Ostrinia furnacalis* to exhibit moderate and low levels of resistance to Cry1Ac and Cry1Aa toxins, respectively [69]. It has also been shown that simultaneous knockdown and knockout of *HaABCB6* can increase the susceptibility of *H. armigery* larvae to gossypol [70].

The polycalin protein has been shown to bind *Bt* toxins in vitro in several studies [43,44,51], including *P. xylostella* [51]. Studies have shown that when *H. armigera* larvae were fed with polycalin antibody and Cry2Aa or Cry1Ac toxins at the same time, the mortality rate of the larvae was significantly lower than that of the group fed Cry2Aa [44] and Cry1Ac [43] toxins alone. Meanwhile, the mortality of *P. xylostella* larvae was also reduced after feeding on the toxin containing the polycalin antibody and Cry1Ac toxin [51]. The most common type of resistance to the *Bt* toxin in Lepidopteran pests is “mode 1”. “Mode 1” resistance must have a high level of resistance to at least one Cry1Ac toxin, be recessive, reduce midgut mucosal binding to at least one Cry1A toxin, and have little or no chance of cross-resistance to Cry1C. However, previous studies have shown that polycalin can bind to both Cry1 and Cry2 toxins. In addition, we found that neither the *Pxpolycalin* gene nor its transcript level were associated with Cry1Ac resistance, but only the *PxABCC2* gene and its transcript level were associated with Cry1Ac resistance, suggesting that polycalin is not a traditional *Bt* toxins receptor and may play a role in *Bt* resistance in insects in another way.

Although *Pxpolycalin* has been confirmed to bind *Bt* toxins *in vitro*, CRISPR/Cas9-based in vivo knockout technology is undoubtedly the most direct evidence to prove whether it plays a role in *Bt* Cry1Ac resistance. ABCC2 is a widely reported *Bt* toxins receptor that has been reported to function in a variety of insects. Recent studies have shown that homozygous strains with simultaneous mutations in several *Bt* toxins receptors obtained by genetic hybridization can develop strong resistance. However, a double mutant strain (Po-C2KO) based on the existing *Pxpolycalin* (PoKO21KI16) and *PxABCC2* (A3KO28) homozygous mutant strains did not show the same synergistic effect (Data Unpublished). Specifically, when treated with 0.5 μg/mL Cry1Ac toxin, the susceptibility of the *Pxpolycalin* and *PxABCC2* genes mutation to Cry1Ac toxin decreased by 22.50% and 86.00%, respectively, compared to the control group (G88); while the susceptibility to Cry1Ac was increased in the strain with those two genes double mutation compared to the strain with just a single *PxABCC2* mutation. It seems that the *Pxpolycalin* gene does not function as a receptor in the *Bt* resistance of *P. xylostella*, but through other pathways, which need to be further investigated.

In conclusion, by qPCR analysis of the transcript levels in the midgut of Cry1Ac-susceptible and -resistant strains, we found that the transcript levels of the *Pxpolycalin* gene were significantly higher in the G88 strain than in the Cry1S1000 strain, and the results of the temporal and spatial expression pattern analysis of this gene in the two strains also showed that the *Pxpolycalin* gene is highly expressed mainly in the larval stage and midgut tissues. In addition, after feeding on an artificial diet containing the Cry1Ac toxin, the expression level of the *Pxpolycalin* gene of *P. xylostella* larvae did not change significantly in a short term and then showed an increasing trend with the increase of larval instar. The results of genetic linkage analysis showed that the *Pxpolycalin* gene and its transcript level were not linked to Cry1Ac resistance, whereas both the *PxABCC2* gene and its transcript levels were linked to Cry1Ac resistance. A single mutation in the *Pxpolycalin* or *PxABCC2* genes mediated by the CRISPR/Cas9 system resulted in decreased susceptibility of *P. xylostella* larvae to Cry1Ac toxin. Specifically, when treated with 0.50 μg/mL Cry1Ac toxin, compared with the control strain G88, the mortality of the homozygous strain with a single mutation of the two genes decreased by 1.29 times and 7.14 times, respectively. In total, our results provide new insights into the potential role of Pxpolycalin and PxABCC2 proteins in Cry1Ac resistance, where PxABCC2 may function as a *Bt* receptor and Pxpolycalin through other pathways, which helps to enrich and refine the resistance mechanism of insects to *Bt* toxins.

## 4. Materials and Methods

### 4.1. Insect Strain and Rearing

The G88 susceptible strain and Cry1S1000 resistant strain [31] of *P. xylostella* were provided by Dr. Anthony M. Shelton in 2016 [71]. G88 was susceptible to the Cry1Ac toxin and Cry1S1000 was resistant to the Cry1Ac toxin. The larvae were fed an artificial diet at a photoperiod of 14 light: 10 dark, 26 ± 1 °C temperature, and 60 ± 5% relative humidity. The adults were given 10% honey water for supplemental nutrition during mating.

### 4.2. Bt toxins and Bioassays

The protoxin Cry1Ac used in this experiment was produced by Btk strain HD-73. Specific purification methods refer to the previous study [72], and the concentration of Cry1Ac was redetermined by the Bradford method (Solarbio, Beijing, China) before each use of the toxin. An artificial diet overlay assay [31] was used to determine the susceptibility of both mutant and wildtype strains of *P. xylostella*. Bioassays were performed on five replicates for seven different concentrations of the Cry1Ac toxin, with an insecticide-free control.

### 4.3. Identification of Sequence Differences between G88 and Cry1S1000 Strains

Based on the predicted *Pxpolycalin* gene sequence of the *P. xylostella* genome database website (http://59.79.254.1/DBM/index.php; accessed on 1 July 2021; *Px005969*) and the *Pxpolycalin* gene sequence in NCBI (MF138149), specific primers were designed and the adult cDNA of *P. xylostella* was used as a template for PCR amplification. Specifically, eight adults from each strain were randomly collected for total RNA extraction. For specific extraction methods, refer to the manual’s instructions ((Eastep Super Total RNA Extraction Kit) Promega, Shanghai, China). FastKing gDNA Dispelling RT SuperMix (TianGen, Beijing, China) was used to synthesize cDNA for subsequent PCR amplification with the reverse transcription program as follows: 42 °C for 15 min and 95 °C for 3 min. Two pairs of specific primers (Poly1F: 5′-CGATTAGTGGTGGCATGGGTG-3′, Poly1R: 5′-CTGCACGTTGTTCAGATCCTGG-3′; Poly2F: 5′-GGGAAACTCAGACAAGCTATTGGG-3′; and Poly2R: 5′-CTAAGCAAATACTCTTTGCATGAGCG-3′) were used to amplify the whole length of *Pxpolycalin* in *P. xylostella* by PCR.

### 4.4. Construction of Phylogenetic Tree

The amino acid sequence encoded by *Pxpolycalin* was compared with other insect proteins on the NCBI website, and the phylogenetic tree was constructed by neighbor-joining (NJ) with 1,000 bootstrap replications using the MEGA 7.0 software.

### 4.5. Spatio-Temporal Expression Patterns of polycalin in P. xylostella

Samples of G88 susceptible and Cry1S1000 resistant strains at different developmental stages (egg, 1st to 4th instar larva, pupa, and male and female adult) and different tissues (midgut, epidermis, head, malpighian tubule, and silk gland) were collected uniformly, and the samples of different tissues were anatomized in PBS. After all the samples were collected, the total RNA was extracted and reversely transcribed into cDNA. Specific primers (qpolycalin-F: 5′-TGGACAGCGTGTCGTATTGCC-3′, qpolycalin-R: 5′-GGAGTGGAAGAGACGTCATAGAAGG-3′) were used to identify the differential expression of *Pxpolycalin* in different developmental stages and tissues [73], and statistically significant differences were analyzed with one-way ANOVA (Tukey’s test for multiple comparisons). The qRT-PCR was conducted on CFX96 (BioRad, Hercules, CA, USA) with a volume of 20 μL (mix 10 μL, 0.4 μL of each primer, RNase free water 7.2 μL, cDNA 2 μL), and the running procedure was as follows: 95 °C for 2 min, followed by 40 cycles of 95 °C for 15 s and 60 °C for 50 s.

### 4.6. Cry1Ac Treatment

To investigate whether the transcription level of the *Pxpolycalin* gene can be induced by the Cry1Ac toxin, their transcript levels in 3rd larvae following exposure to an LC_10_ (0.10 μg/mL) dose of the Cry1Ac toxin were determined. According to the present research, the induction of the *Pxpolycalin* gene in response to the Cry1Ac toxin dissolved in PBS was determined by treating the early 3rd instar of the G88 strain. Five time points (6 h, 12 h, 24 h, 36 h, 48 h) after the Cry1Ac treatment were used to examine the effect on the *Pxpolycalin* gene. Larvae treated with PBS were used as the control. At each time point, four × 10 surviving larvae treated with Cry1Ac toxin or PBS were collected, removed quickly to liquid nitrogen, and stored at −80 °C until RNA extraction.

### 4.7. Genetic Linkage Analysis

**Figure 9:**
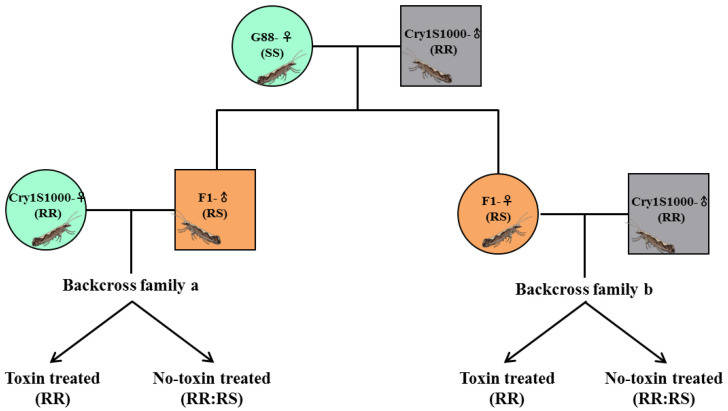
Diagram showing the genetic linkage analysis strategy of Pxpolycalin with Cry1Ac resistance. A female G88 (susceptible) was crossed with a male Cry1S1000 (resistant) to produce F1 progeny. Next, the hybrid F1 was backcrossed with Cry1S1000. Half of the backcross family a and b were treated with 0.5 μg/mL Cry1Ac toxin, and the other half together with the surviving larvae/adults from the treated group were sampled directly.

The G88 and Cry1S1000 strains were used for genetic linkage analysis and the hybridization strategy is shown in Figure 9. In simple terms, the hybrid F_1_ generation produced by the mating of the male Cry1S1000 adult [67,74] and female G88 adult was backcrossed with the Cry1S1000 resistant strain. Half of the backcross family a and b were treated with 0.5 μg/mL [31] Cry1Ac toxin, and the other half together with the surviving larvae/adults from the treated group were sampled directly. For linkage analysis between the expression levels of *Pxpolycalin* and Cry1Ac resistance, we tested *Pxpolycalin* transcript levels in each larva from F_1_, backcrossed a and b of the Cry1Ac toxin both treated and untreated by qPCR, as described above. For linkage analysis between the *Pxpolycalin* gene and Cry1Ac resistance, a 340-bp/338-bp gDNA fragment of *Pxpolycalin* was amplified from each adult of the backcross family a and b by PCR cloning with a pair of gene-specific primers (gPolycalin-F: 5′-TGGCTAGCGATGATAACAGTGC-3′, gPolycalin-R: 5′-CTGCACGTTGTTCAGATCCTGG-3′); (gABCC2-F: 5′-CATGGCTACTGCTACTACG-3′, gABCC2-R: 5′-CTTTCCAATGAAACCAAC-3′) and the amplicons were subsequently sequenced to distinguish the resistant- and susceptible-allele types.

### 4.8. Preparation of sgRNA and Cas9 Protein

According to the principle of 5′-N20NGG-3′ (with the PAM underlined), the recognition site of sgRNA was designed on the exon 2 of the *Pxpolycalin* gene, and the potential off-target effect was analyzed by Cas-OFFinder (http://www.rgenome.net/cas-offinder/; accessed on 16 May 2022). KOD-401 (TOYOBO, Osaka, Japan) was used for PCR amplification in vitro, and the reaction system was 200 μL: 4 μL KOD-Plus-Neo, 20 μL 10 × PCR Buffer, 20 μL 2 mM dNTPs, 12 μL 25 mM MgSO_4_, 6 μL upstream and downstream primers (sgRNA-F: 5′- TAATACGACTCACTATAGGCACCCCCAGTGCGAGTGAGCGTTTTAGAGCTAG-3′, sgRNA-R: 5′-AAAAGCACCGACTCGGTGCCACTTTTTCAAGTTGATAACGGACTAGCCTTATTTTAACTTGCTATTTCTAGCTCTAAAA-3′), and 132 μL ddH_2_O. The reaction procedure is as follows: 98 °C for 2 min, 35 cycles at 98 °C for 10 s, 55 °C for 30 s, and 68 °C for 30 s. At 68 °C, the PCR amplification products were extended for the final 5 min. Finally, the PCR amplification products were recycled with the gel extraction kit (Omega, Morgan Hill, GA, USA), and the recycled products were the sgRNA in vitro transcription template. In vitro transcription of sgRNA was performed using the HiScribe T7 Quick High Yield RNA Synthesis Kit (New England Biolabs, Ipswich, MA, USA), in which 250 ng of template was used, and DNase I was added after the reaction to remove the DNA template. Then, the method of phenol: chloroform: isopentyl alcohol was used to purify sgRNA. After the quality of the purified RNA was confirmed by electrophoresis, it was stored at −80 °C, and the concentration was redetermined before each use. The Cas9 protein used in this experiment was purchased from the GeneScript Corporation (GeneScript, Nanjing, China).

### 4.9. Microinjection of P. xylostella Embryos and Screening of Homozygous Strains

**Figure 10:**
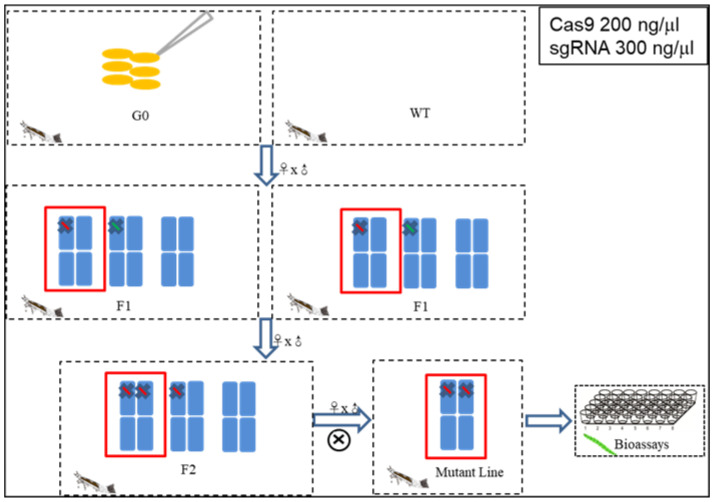
The strategy of homozygous mutant line construction. The unmated adult of the G_0_ generation was hybridized with the G88 strain, and the gDNA of the adult G_0_ was extracted after oviposit. Then, the mutant was detected by PCR sequencing, and the offspring of the same mutation type was selected to obtain homozygous mutant line.

The mixture containing 200 ng/μL Cas9 protein and 300 ng/μL sgRNA was injected into fresh eggs produced within 15 min using the IM 300 Microinjector (Narishige, Tokyo, Japan) mounted on the SZX16 Stereo Microscope (Olympus, Tokyo, Japan). The surviving G_0_ generation was screened for mutant lines according to the homozygous mutant line screening strategy as shown in Figure 10, that is, the injected adults of the G_0_ generation were crossed with the G88 strain, and G_0_ adult gDNA was extracted using TIANamp Genomic DNA Kit (Tiangen, Beijing, China) after spawning, and the mutant type was detected by sequencing (Polycalin-f: 5′-TTCACATCTACAACATGGAACGAGG-3′, Polycalin-r: 5′-CATTGCTCGTGAGTTTAGTAGG-3′). The offspring of the same mutation type was further selected to obtain the homozygous mutant line.

## Figures and Tables

**Table 1:** Genetic linkage of the *R_Po_* allele with Cry1Ac resistance.

Generation	N ^a^	Genotype ^b^	
		*R_Po_S_Po_*	*R_Po_R_Po_*
F_1_ (G88 ♀ × Cry1S1000 ♂)	10	10	0
Backcross family a with toxin treated	12	9	3
Backcross family a without toxin treated	24	11	13
Backcross family b with toxin treated	12	3	9
Backcross family b without toxin treated	24	12	12

^a^ Total number of adults used for genotyping. ^b^ For the genetic linkage analysis, we used Fisher’s exact test. The observed genotype frequencies of *Pxpolycalin* on a diet treated with the Cry1Ac toxin were not significantly different from the expected genotype frequencies on the untreated diet (*χ^2^* = 2.76, *df* = 1, *p* > 0.05 for Backcross family a; *χ^2^* = 2.06, *df* = 1, *p* < 0.05 for Backcross family b).

## Data Availability

Sequence data of Pxpolycalin have been deposited in the National Center for Biotechnology Information. All other relevant data are included in the main text and Supplementary Materials.

## Supplementary Materials

The following supporting information can be downloaded at: https://www.mdpi.com/article/10.3390/toxins15040273/s1, Figure S1. *R_A2_* mutation of *PxABCC2* from the Cry1S1000 strain. Alignment of gDNA sequences of the *S_A2_* allele and the *R_A2_* allele. Sequences highlighted in orange are the exons 6 and 7; Figure S2. Analysis of the linkage between the resistance to Cry1Ac and reduced *Pxpolycalin* (**A**) and *PxABCC2* (**B**) expression levels in the Cry1S1000 strain of *P. xylostella*. Expression levels of *PxABCC2* and *Pxpolycalin* in individual midgut from larvae of the F_1_, without Cry1Ac-selected (untreated) and with Cry1Ac-selected (treated) backcross families (backcross family a and b) are shown relative to levels in the susceptible (G88) strain; Figure S3. Mutagenesis of *PxABCC2* induced by CRISPR/Cas9. Partial sequences from the G88 and homozygous *PxABCC2* mutant showing the indels at the target sequence (ABCC2-sg2) in exon 3 of *PxABCC2*; Table S1. Genetic linkage of the *R_A2_* allele with Cry1Ac resistance.
