# Supplementary material for: Analysis of the Effect of Plutella xylostella Polycalin and ABCC2 Transporter on Cry1Ac Susceptibility by CRISPR/Cas9-Mediated Knockout
Source: Toxins (Basel). 2023 Apr 6;15(4):273. doi: 10.3390/toxins15040273 (PMC10145054; doi:10.3390/toxins15040273)
Supplement: Supplementary file 1 [file toxins-15-00273-s001.zip › toxins-2242695-supplementary.pdf]

# Supplementary Materials

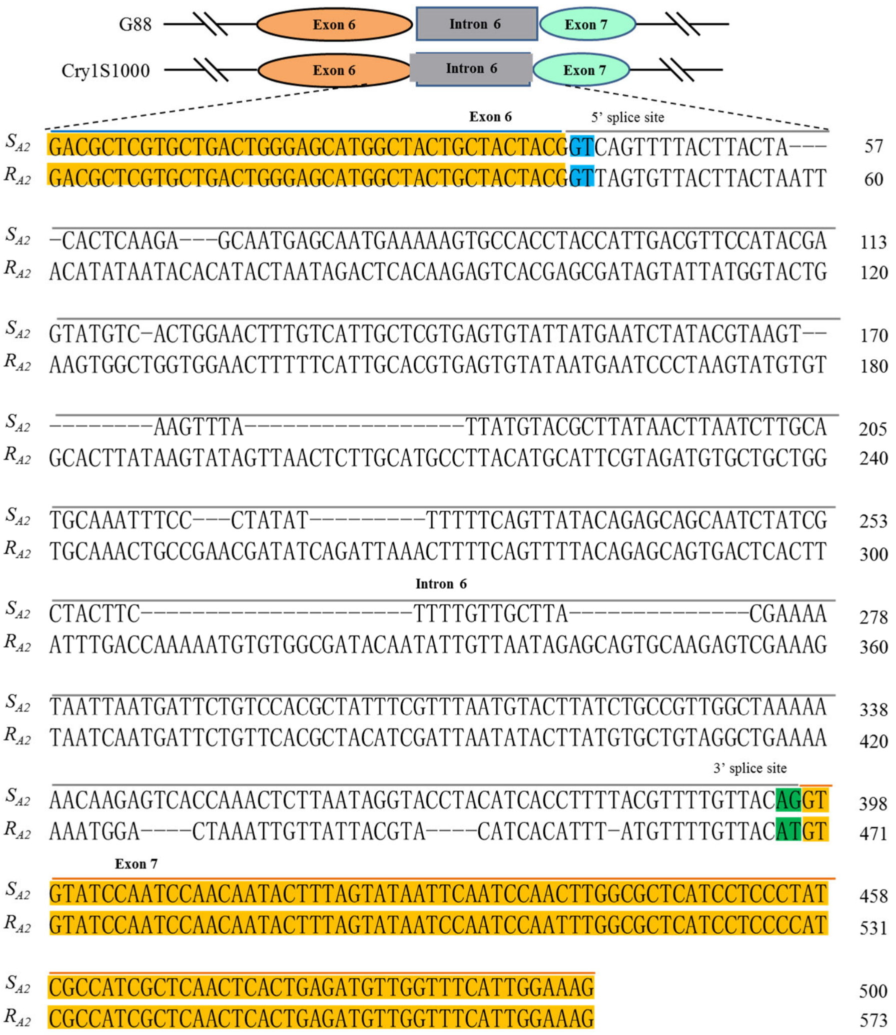


**Figure S1.** *$R_{A2}$* mutation of *PxABCC2* from the Cry1S1000 strain. Alignment of gDNA sequences of the *$S_{A2}$* allele and the *$R_{A2}$* allele. Sequences highlighted in orange are the exons 6 and 7.

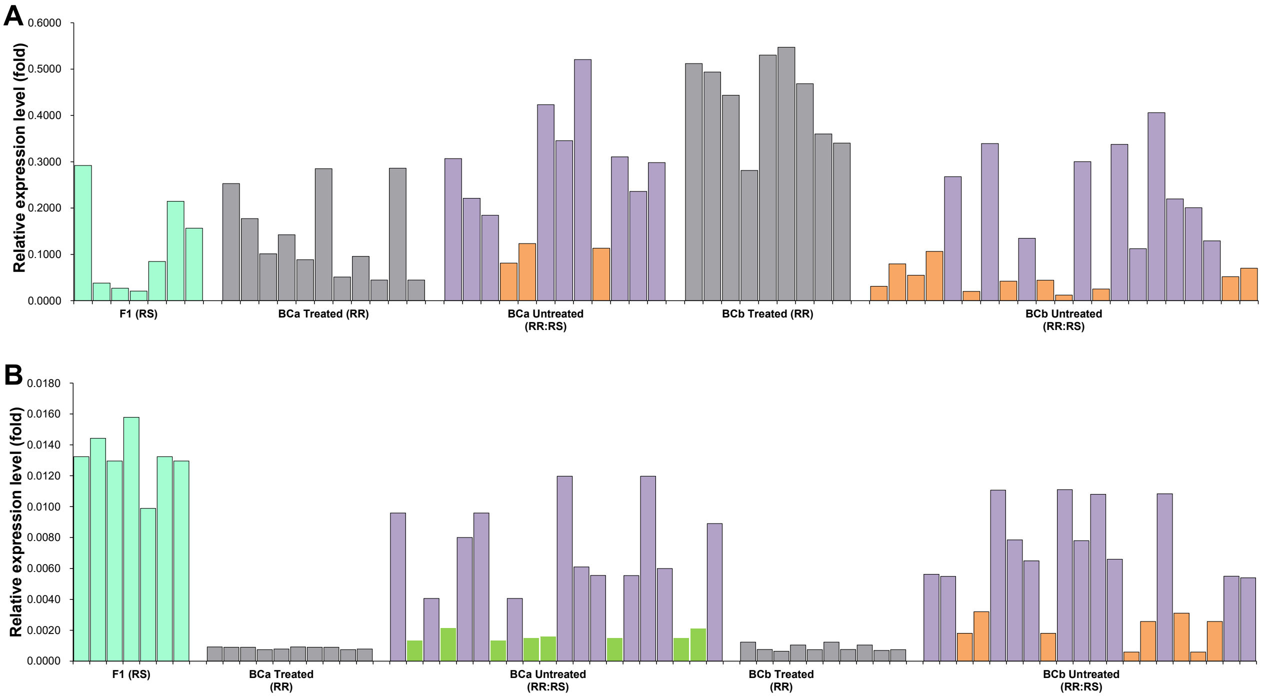


**Figure S2.** Analysis of the linkage between resistance to Cry1Ac and reduced *Pxpolycalin* (A) and *PxABCC2* (B) expression levels in the Cry1S1000 strain of *P. xylostella*. Expression levels of *PxABCC2* and *Pxpolycalin* in individual midgut from larvae of the $F_1$, without Cry1Ac-selected (untreated) and with Cry1Ac-selected (treated) backcross families (backcross family a and b) are shown relative to levels in the susceptible (G88) strain.

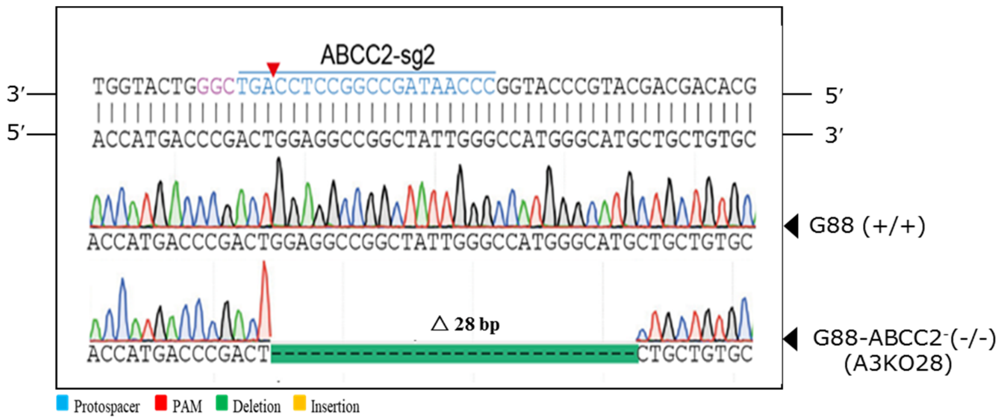


**Figure S3.** Mutagenesis of *PxABCC2* induced by CRISPR/Cas9. Partial sequences from the G88 and homozygous *PxABCC2* mutant showing the indels at the target sequence (ABCC2-sg2) in exon 3 of *PxABCC2*.

**Table S1.** Genetic linkage of the $R_{A2}$ allele with Cry1Ac resistance.

| Generation | N[a] | Genotype[b] | |
|---|---|---|---|
| | | $R_{A2}S_{A2}$ | $R_{A2}R_{A2}$ |
| $F_1$ (G88♀ x Cry1S1000♂) | 10 | 10 | 0 |
| Backcross family a with toxin treated | 12 | 0 | 12 |
| Backcross family a without toxin treated | 24 | 14 | 10 |
| Backcross family b with toxin treated | 12 | 0 | 12 |
| Backcross family b without toxin treated | 24 | 15 | 9 |

[a] Total number of adults used for genotyping.

[b] For the genetic linkage analysis, we used Fisher's exact test. The observed genotype frequencies of *PxABCC2* on diet treated with Cry1Ac toxin differed significantly from the expected genotype frequencies on the untreated diet ($\chi^2 = 11.46$, $df = 1$, $P < 0.001$ for Backcross family a; $\chi^2 = 12.86$, $df = 1$, $P < 0.000$ for Backcross family b).
